# Analysis of Social Determinants of Health and Disability Scores in Leprosy-Affected Persons in Salem, Tamil Nadu, India

**DOI:** 10.3390/ijerph15122769

**Published:** 2018-12-06

**Authors:** Martin Heidinger, Elisa Simonnet, Sr. Francina Karippadathu, Markus Puchinger, Johann Pfeifer, Andrea Grisold

**Affiliations:** 1 Global Health and Development, Medical University of Graz, Neue Stiftingtalstraße 6, 8010 Graz, Austria; elisa-simonnet@orange.fr (E.S.); johann.pfeifer@medunigraz.at (J.P.); andrea.grisold@medunigraz.at (A.G.); 2 Doctor Typhagne Memorial Charitable Trust, S.M.M.I. Convent Staff Quarters, St. Mary’s Hospital Campus, Arisipalayam, Salem, Tamil Nadu 636009, India; dtmctrust@gmail.com; 3 Research Unit for Medical Engineering and Computing, Medical University of Graz, Auenbruggerplatz 29, 8036 Graz, Austria; markus.puchinger@medunigraz.at; 4 Department of General and Visceral Surgery, Medical University of Graz, Auenbruggerplatz 29, 8036 Graz, Austria; 5 Institute of Hygiene, Microbiology and Environmental Medicine, Medical University of Graz, Neue Stiftingtalstraße 6, 8010 Graz, Austria

**Keywords:** leprosy, India, disability, EHF score, social determinants of health, multiple stepwise linear regression analysis

## Abstract

A consistent relationship has been found between leprosy and inequities in social determinants of health. It, however, remains unclear which aspect of these social determinants contributes most to the risk of infection, and even less clear are the risk factors for the development of leprosy-related disabilities. The objective of this study was to elicit the differential impact of social determinants of health in leprosy-affected persons, and determine whether structural inequities in accessibility to societal resources and lower socioeconomic parameters correlated with higher severity of disabilities. This analysis was based on a sampled population affected by leprosy in Salem, Tamil Nadu, India. Persons enrolled in the study were covered by a nongovernmental lifelong care program, had completed a multidrug therapy for leprosy and/or were slit-skin-smear negative, and showed Grade 1 or higher disabilities due to leprosy. Multiple stepwise linear regression analysis was performed. The Eyes-Hands-Feet (EHF) score was the outcome variable, and gender, age, time after release from treatment, monthly income, and living space were explanatory variables. There were 123 participants, comprised of 41 (33.33%) women and 82 (66.67%) men. All study participants belonged to India’s Backward classes; 81.30% were illiterate and the average monthly income was 1252 Indian rupee (INR) (US$19.08 or €17.16). The average EHF score was 7.016 (95% CI, 6.595 to 7.437). Stepwise multiple linear regression analysis built a significant model, where *F*(2, 120) = 13.960, *p* ≤ 0.001, effect size (Cohen’s f2) = 0.81, explaining 18.9% of the variance in EHF scores (*R*^2^ = 0.189). Significant predictors of a higher EHF score in persons affected by leprosy were found to be higher age (beta = 0.340, 95% CI, 0.039 to 0.111, *p* < 0.001), as well as less living space (beta = −0.276, 95% CI, −0.041 to −0.011, *p* = 0.001). Our results suggest that inequalities in social determinants of health correspond to higher disability scores, which indicates that poor living standards are a common phenomenon in those living with leprosy-related disabilities. Further research is needed to dissect the exact development of impairments after release from treatment (RFT) in order to take targeted actions against disability deterioration.

## 1. Introduction

Leprosy is a chronic, granulomatous infectious disease caused by *Mycobacterium leprae* [1]. A consistently strong relationship between leprosy and inequities in social determinants of health has been found [2,3,4,5,6]. These social determinants of health comprise the sociopolitical context as well as structural mechanisms that shape social hierarchies, and result in an individual’s socioeconomic position [7]. However, it is not yet clear whether the sociopolitical context (e.g., public policies), structural mechanisms (e.g., poverty through rigid social hierarchies), and/or the individual’s overall socioeconomic position contribute most to the risk of infection [3,4,6,7,8]. While persons with disabilities were shown to experience social- and health-status disparities [9,10,11,12,13], the relationship between disabilities due to leprosy, and social determinants of health remains unclear.

In 2016, over 95% of new leprosy cases were diagnosed in 22 global priority countries, as defined by the World Health Organization (WHO). India, Brazil, and Indonesia were the top contributors. India globally accounts for over 60% of newly diagnosed leprosy cases, and over four-fifths of the WHO’s new South-East Asian Region’s (SEAR) diagnosed cases [14]. In the reporting period of 2016/17, Tamil Nadu, the state in which the present study was conducted, showed a prevalence rate (PR) of 0.41 per 10,000 people and an annual new-case detection (ANCD) rate of 6.27 per 100,000 people [15]. Salem, the district in which the investigation took place, showed a PR of 0.32 per 10,000 people as of March 2015, and an ANCD of 4.80 per 100,000 people in the reporting period 2014/15 [16].

India has a social structure that divides society into classes. Backward classes comprise of Scheduled Tribes (ST), Scheduled Castes (SC), and Other Backward Classes (OBC). They can be defined as marginalized and disadvantaged groups [17]. Backwardness consists of a lack of adequate opportunities for individual social, economic, and/or educational development [17,18]. STs and SCs are constitutionally defined as communities that are scheduled according to Articles 341 and 342 of India’s Constitution, respectively [19,20,21]. Articles 341 and 342 state that the president can, by public notification, specify the communities that are deemed as STs or SCs [19]. OBCs are defined as any other backward class, other than ST and SC, which is regulated by the National Commission for Backward Classes Act [22]. In Tamil Nadu, OBCs are further divided into Backward Classes (BC) and Most Backward Classes (MBC) [23]. Over one-third of India’s newly detected cases were attributed to persons who were members of a ST or SC [24]. While OBCs and other lower parts of India’s society were not explicitly depicted in the report of the National Leprosy Elimination Program (NLEP), the correlation between socioeconomic status, the pattern of transmission and infection, as well as the probability of successful treatment, is nonetheless an important consideration [5,25,26].

Moreover, viable *M. leprae* was recently found in soil and water [27,28]. Red squirrels in the British Isles were also found to form a reservoir for leprosy bacilli [29]. Therefore, the possibility of yet undiscovered host diversity is crucial for future disease-control measures [30].

Disabilities in leprosy are graded by the WHO’s leprosy-disability grading system into Grades 0, 1, and 2 for the number of eye, hand, and foot impairments, as depicted in Table 1 [31]. Only rough estimates exist regarding the global burden of disabilities due to leprosy, with numbers suggesting as many as three million affected people [32]. In 2016, 12,819 leprosy cases with Grade 2 disabilities at diagnosis (G2D) were found globally. This was 5.9% of all newly detected cases in 2016, and translates to an incidence rate of 1.7 cases per million persons [14]. Globally, the number of G2D-affected leprosy patients has shown a remote decrease over the last decade [14]. Over the same time period, however, cases increased in the SEAR, as well as on a national level in India. The SEAR currently accounts for 58% of all global G2D cases. In 2006, 6332 new cases were detected with Grade 2 disabilities in the SEAR [33], increasing by 16.84% to 7398 in 2016, and resulting in a current G2D rate of 3.8 per million people [14]. This rise has been even more significant in India, from 3130 G2D cases in 2006 [33] to 5245 in 2016, increasing by just over two-thirds (67.57%). However, when comparing the reporting periods of 2015 and 2016, a marked decrease of more than 10% on both the regional and national levels could be found [14]. The national rate of G2D cases in India was 2.9 G2D cases per million people in 2016 [14]. In absolute numbers, Tamil Nadu registered 199 new G2D cases in 2016, showing a G2D rate of 2.53 per million people [15]. Salem had 17 G2D cases in 2015 and, therefore, a rate of 4.6 per million people [34]. Global data regarding the rate of Grade 1 disabilities at diagnosis (G1D) in leprosy, and the development of impairments, are not available. Only very limited information exists concerning the development of Eyes-Hands-Feet (EHF) scores during the lifespan of affected persons [35].

The objective of this study was to elicit the differential impact of social determinants of health in leprosy-affected persons, and to determine whether structural inequities in access to societal resources [36] and lower socioeconomic parameters correlate with higher severity of disabilities in persons affected by leprosy.

## 2. Materials and Methods

### 2.1. Study Area

The study was performed based on a long-lasting cooperation between the Doctor Typhagne Memorial Charitable (DTMC) Trust, Salem, Tamil Nadu, India, and the Medical University of Graz, Austria. The DTMC Trust is a nongovernmental, charitable institution that focuses its work on the diagnosis, treatment, and lifelong care of persons affected by leprosy, tuberculosis, and the human immunodeficiency virus. Since 1981, the DTMC Trust has been integrated into the NLEP [37]. Salem District, the district in which the present study was conducted, located in the state of Tamil Nadu, has roughly 3.5 million inhabitants, where 54% live in rural areas and 19% as part of STs and SCs, with an overall literacy rate of 74% [38].

### 2.2. Study Population

The inclusion criteria targeted cured persons affected by leprosy who were released from treatment (RFT), and were part of the DTMC Trusts lifelong-care program in the Salem District. Requirements were a completed leprosy multidrug-therapy (MDT) regimen, or a negative slit-skin-smear result in those with dapsone-only treatment before introducing the MDT. Additionally, disabilities due to leprosy of Grade 1 or higher had to be present in the eyes, hands, and/or feet in order to be recorded. Participants were required to be aged 18 years or older, and geographical availability (i.e., accessible areas, drivable roads, etc.) as well as personal presence given.

### 2.3. Data Collection

**Figure 1:**
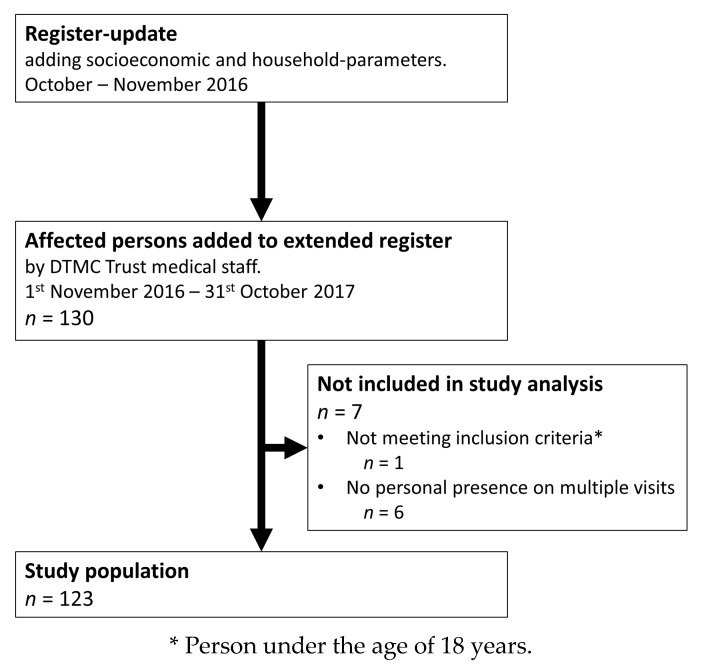
Flow diagram of the study and included leprosy-affected persons from the updated register of the Doctor Typhagne Memorial Charitable (DTMC) Trust.

An extension of the local register regarding the lifelong-care of disabled persons due to leprosy was conducted in October and November 2016, in Salem, Tamil Nadu, India. This broadened the range of socioeconomic status among participants, as well as other indicators of the condition within each household. Persons who were part of the lifelong-care program of the DTMC Trust, that were visited during routine annual follow-up visits from 1 November 2016 to 31 October 2017, were added to the extended register using a convenience-sampling technique (*n* = 130). Of these, seven did not meet the inclusion criteria and were therefor excluded from the study population, resulting in a final study population of 123 participants, as depicted in Figure 1.

The survey for each patient covered demographic, economic, household, and disability parameters. Participants were categorized by social class as per national and statewide classifications [39,40,41]. Furthermore, the participant’s status regarding a monthly deformity pension (DP) from the Indian National Government of 1000 INR (US$15.24 or €13.71) per month was followed up. Status of the household was also included, which consisted of ownership status, the number of persons living together, the size of their respective living spaces, as well as the measurement of distance to certain points of interest. Deformity history was obtained, and disabilities were evaluated according to Brandsma’s Operational Guidelines of the WHO’s disabilities grading for eyes, hands, and feet, as presented in Table 1 [31]. Participants were inspected and tested as recommended with a standard operating procedure [31]. This included eyelid-strength and vision testing at a distance of 6 meters. Sensory impairment of the extremities was tested with a ballpoint pen. The following testing sites were used for the hands: Distal pulp of digit V and hypothenar eminence, distal pulp of digits I and II, and thenar eminence. Testing sites of the feet were digit I, the 1st and 5th metatarsal head, and the midlateral border of the foot. In public health terms, the WHO’s maximum grading (which classifies the disability status of a person via their maximum scoring on any of the testing sites, as depicted in Table 1) prevails. However, the EHF sum score, which is the sum of each disability score (from 0 to 2 points) of the 6 sites investigated (each eye, hand, and foot), resulting in a score ranging from 0 to 12 points, represents a reliable scoring system of leprosy disabilities and a potentially more sensitive tool to monitor disability changes and hidden disabilities compared to the WHO’s maximum grading [42,43,44,45]. For this reason, we used this scoring system in our analysis. After the investigation of each testing site, the EHF score was calculated.

The anonymized dataset is included in Supplementary File S1—Dataset. A STROBE Statement concerning cohort studies can be found in the Supplementary Materials (Checklist S2—STROBE Statement).

### 2.4. Data Analysis

Normal distribution of the data was verified with the Kolmogorov–Smirnov test. Patient characteristics of the study population are presented as descriptive statistics. Significant predictors were determined via stepwise multiple linear regression analysis, with the EHF score as the dependent variable, and the following as explanatory variables: Gender, age, time after release from treatment, monthly income, and living space. Analysis of variance (ANOVA) was used to determine the statistical significance of *F* values. The level of significance was set at an alpha value of *p* = 0.05. The software package used was IBM^®^ SPSS^®^ Statistics V25.0 (New York, NY, USA).

The currency-exchange conversions used in this study were taken from the International Monetary Fund’s Online Exchange-Rate Wizard, and represent average exchange rates from 1 November 2016 to 31 October 2017.

### 2.5. Ethical Considerations

Approval for the study and the presented methodology was granted by the ethical review committee of the Medical University of Graz, Austria (29–578 ex 16/17). Informed consent was obtained from all study participants in written form if they were literate, otherwise orally after direct translation to the local language and documentation of their approval via fingerprint. All study participants were reassured that nonparticipation would not affect their care.

## 3. Results

### 3.1. Demographic Data

A total of 123 patients were included in the study, with 41 (33.33%) women and 82 (66.67%) men. Their age ranged from 27 to 95 years, with a mean of 65 years. The number of persons who stated they were married was 85/123 (69.11%). However, one-fifth of these, or 17/123 (13.82%), were found to be living by themselves; only 55.28% of participants (*n* = 68) shared a home with their spouse. Of the remaining participants, 19/123 (15.45%) declared themselves unmarried, and 19/123 (15.45%) were widowed. Table 2 summarizes the information regarding demographic, economic, household, and disability situations.

### 3.2. Economic Situation

The average monthly income of the total study population was 1252 INR, which was equivalent to €17.16 or US$19.08. For the 68 participants living with their spouse, the average monthly income was 1434 INR. Their partners had an average income of 740 INR over the same period, resulting in a monthly household total of 2174 INR. The participants’ average monthly income was mainly through provision of the deformity pension from the Indian National Government, which 81.30% (100/123) of the participants received. Ninety-two of the 123 participants (74.80%), and 29/47 (61.70%) below the age of 65 years, indicated that they were unemployed. Of the participants who were employed, 18/31 (58.06%) depended on seasonal day work, 11/31 (35.48%) were self-employed, while two out of 31 (6.45%) had a permanent position. The average monthly income for the employed study participants was 1782 INR (€24.43 or US$27.16).

### 3.3. Household Situation

Most participants (74.80%) were living in their own house, while 3/123 (2.44%) were homeless. Living spaces were 28.7 m^2^ on average and ranged from 4–140 m^2^ (excluding homeless participants). The number of people per household ranged from one to nine persons, resulting in an average floor area per person of 11.03 m^2^.

One hundred and twenty out of 123 (97.56%) participants had no water source inside their house. Ninety-one out of 123 (73.98%) regularly defecated in the open. Cooking was carried out by 62/123 (50.41%) over open fire, 51/123 (41.46%) were using gas stoves, and two out of 123 (1.63%) persons had electric stoves. Eight out of 123 (6.50%) persons did not cook themselves but had access to a central food dispensary.

### 3.4. Disabilities

Disability scores based on the WHO’s maximum grading in participants with a monthly income of ≤1000 INR compared to participants with >1000 INR per month are presented in Table 3. Site-specific grading showed that the majority had Grade 2 disabilities on both hands or both feet, and that 51.22% had Grade 2 disabilities on both hands and both feet.

The EHF score average was 7.016, ranging from 1–12. An EHF score below seven was found in just over one-third of cases, as shown in Table 3. A score higher than nine was found in 15/123 (12.20%) participants.

### 3.5. Regression Analysis

Stepwise multiple linear regression analysis built a significant model, where *F*(2, 120) = 13.960, *p* ≤ 0.001, effect size (Cohen’s f2) = 0.81, explaining 18.9% of the variance in EHF scores (*R*^2^ = 0.189). The adjusted *R*^2^ was found to be equal to 0.175. Analysis of variance inflation factors (VIFs) did not demonstrate multicollinearity between factors, as depicted in Table S1. No violations of linearity were detected. Significant predictors of a higher EHF score in persons affected by leprosy were found to be higher age (Figure S1a) as well as less living space (Figure S1b), as shown in Table 4.

Generally, women (6.976, 95% CI, 6.396 to 7.555) had a slightly lower average EHF score than men (7.073, 95% CI, 6.468 to 7.605); however, gender was excluded as a non-significant predictive variable for the EHF score (beta = 0.009; *p* = 0.912). Nevertheless, more men showed an EHF score above nine (14.63%) than women (7.32%).

Monthly income showed significant negative correlation in Pearson’s correlation (−0.076, *p* = 0.026). However, it was excluded as a non-significant predictive variable (beta = −0.078; *p* = 0.360).

Data stratification according to the time since RFT saw limitations due to a lack in exact monitoring prior to MDT regimens. Therefore, patients with no record (*n* = 27) were included with 1988 as the completion year. This assumption was made based on the introduction of MDTs in 1986 in Tamil Nadu, with the longest regular therapeutic regimen taking one year. Time since RFT showed a significant positive correlation in Pearson’s correlation (0.199; *p* = 0.014). However, it was excluded as a non-significant predictive variable for the EHF score (beta = 0.118; *p* = 0.174).

## 4. Discussion

Leprosy has long been considered a disease in which political, economic, and structural or more general social inequalities correlate with the risk of transmission and infection, as well as the probability of proceeding to a successful cure [2,3,4,25,26,46]. As a Neglected Tropical Disease (NTD), it may serve as a reflection of health inequities within a society, as NTDs tend to exhibit the interconnection of poverty-related pathologies and neglected populations [47]. Marginalized persons must therefore be a subject of particular focus, as it is their living conditions, motivation, and understanding that determine early reporting, treatment compliance, and the actions of lifelong care [25,26]. In this cross-sectional study, which included persons affected by leprosy who were RFT, showed disabilities due to *M. leprae* and were part of a lifelong care program of a nongovernmental institution in the district of Salem, Tamil Nadu, we undertook descriptive as well as multiple stepwise linear regression analyses to provide information about the socioeconomic status, structural characteristics, and the disability status of the affected persons.

The population can be summarized as poorly educated and of low socioeconomic status. Moreover, social class distribution seems especially noteworthy, as all study participants belonged either to SCs, STs, or OBCs, constituting the lowest parts of India’s social hierarchy, and none was a member of the forward classes, showcasing structural mechanisms in pathogenesis and disease progression. The caseload of persons from SCs and STs, which makes up more than one-third (37.36%) of the national annual new caseload, and 21.99% in Tamil Nadu, is the only one India’s NLEP is highlighting in its records today [24]. Therefore, these persons, together with those belonging to OBCs, can be prioritized as those who require particularly long and intensive post-MDT follow-ups, self-care instructions, and support. The high rate of illiterate participants shows the immediate demand for increased visual information rather than written guidance.

As defined by the World Bank Group, those who live in extreme poverty include people living on less than US$1.90 (€1.71) per day [48,49]. Based on the average income of the surveyed persons, people affected by leprosy live well below this rate, with US$0.63 (€0.56) on average, and US$0.89 (€0.80) per day for employed study participants. Additionally, the latter often depend on adequate climate conditions, e.g., for agricultural work, and employer goodwill or nescience regarding their health status. Along with the high rate of unemployed persons in this study population, even in those below 65 years of age, extreme poverty highlights the socioeconomic challenges of leprosy-affected persons, which have already been addressed by governmental support, showcased in the high number of deformity-pension recipients. However, out-of-pocket health expenditure was found to be increased in disabled persons with low income [50]. The economic burden of leprosy was recently elicited by Tiwari et al., linking patients’ expenditures in primary care to the strength of public health services [51]. Even though our results showed a monthly income that was excluded as a non-significant predictive variable for higher disabilities, the above-mentioned results stress the need for a continued comprehensive approach to social and work reintegration for persons affected by leprosy.

Our results underscore the low housing standards in which leprosy-affected persons live today, as well as the significant predictive value that less living space has on higher disabilities due to leprosy (Figure S1b). The U.S. Department of Housing and Urban Development proposes a cut-off of 165 square feet (equaling 15.33 m^2^) average living space per person as the definition for overcrowding [52]. In our population, 91/123 (73.98%) participants had less than the proposed minimum living space. Almost all participants had to fetch water outside of their house, and the rate of open defecation is problematic as a culturally deep-rooted habit of Indian people that poses hygiene issues, as indicated by a recent United Nations International Children’s Emergency Fund (UNICEF) report [53].

Whether disabilities due to leprosy are progressing throughout the life of an affected person has not been thoroughly investigated. Saunderson et al. showed a correlation between episodes of neuropathy after diagnosis, deterioration of disabilities, and an increase of individual EHF scores in studies of the ALERT MDT Field Evaluation Study (AMFES) cohort [35]. The impairment status at MDT initiation was found to be the most important determinant for future impairment, and impairment dynamics were less favorable after RFT [54]. De Oliveira et al. showed that, among patients without impairments at diagnosis, 5% of paucibacillary (PB) and 20% of multibacillary (MB) cases developed disabilities throughout the course of treatment. The majority of both PB and MB cases remained on their respective disability grading from diagnosis until RFT [55]. In this analysis, we also included patients from the dapsone-treatment era. When MDT was introduced, all of these patients had to either provide a negative slit-skin smear or were provided with a 12-month course of MDT before RFT. All patients were successfully treated for *M. leprae* infections, but the development of disabilities throughout the life of affected persons, especially under dapsone-only treatment, remains unclear. Our results show that higher age was a significant predictor of higher disability scores (Figure S1a), but no significant correlation could be found with respect to the time after RFT.

In this regard, the “model of the social production of disease” by Diderichsen et al., elaborates the pathway from an individual’s social position, through exposure to causes of diseases, to the social and economic consequences of acquired diseases and injuries. It shows the relationship of the societal level, including its policy context and social context, with the individual level [56]. Considering the above-mentioned social inequalities and structural inequities in access to societal resources in our study population and Diderichsen’s model, only a comprehensive, integrative policy-making approach will succeed in addressing the societal as well as the individual level in order to reduce inequalities [7,57]. In our regression analysis, we could identify two structural determinants of health, namely, higher age and less living space, as highly significant predictors of higher EHF scores. These explanatory variables built a highly-significant linear regression model in our exploratory study, showing an acceptable yet rather weak effect size (*R*^2^ = 0.189 and *R*^2^ adjusted = 0.175) without multicollinearity (Table S1).

Therefore, the WHO’s second pillar of its Leprosy Strategy 2016–2020, which aims to reduce G2D [58], protects a very vulnerable group of persons who, with adequate measures taken, might be able to remain at their non-existent or at least non-disfiguring disability status. This should help to sustainably reduce the global disease burden due to leprosy, as well as the discrimination against affected persons. As shown in the WHO’s 2017 leprosy report, G2D cases have been reduced for the first time within the last nine years. Even though more cases were found, G2D cases were reduced by over 10% in the SEAR and on a national level [14]. However, the remaining global rate of G2D, as well as in more defined geographical areas, shows that patients are still reporting late, and are diagnosed at a late stage of the disease. Interestingly, it was the Salem District that showed the highest G2D rates per million on all levels of analysis presented in our introduction. As has been shown by Madhavan et al. in an earlier report for the district of Salem [59], our presented population also showed striking rates of Grade 2 disabilities. Almost two-thirds had Grade 2 disabilities of both hands or both feet, leaving the affected person in a desperate state of disability and disfigurement. Eye disability rates were much lower than for hands and feet, but discrimination of Grade 1 disabilities was much more delicate than for hands and feet. Analysis of vision within a population with poor general eyesight analysis and vision adjustment could only in very few cases deliberately be attributed to leprosy. In order to continue to decrease the stigma of the disease and, therefore, increase early reporting while decreasing G2D, currently disabled persons, as well as those developing disabilities throughout the course of treatment or after RFT, ought not to be forgotten, as G2D is also partly due to neglected paresthesia, ulcers, and disfigurements.

### Study Limitations

The present data showed a local snapshot rather than a long-term evaluation of functioning care by the Indian Health Authorities. Parameters were also limited to the presented number of participants, which should ideally be augmented for further investigations. These were added to the extended register of the DTMC Trust that, as a nongovernmental charitable provider of lifelong care in Salem, Tamil Nadu, poses possible selection bias per se. Furthermore, the limitations of a convenience-sampling technique apply. Limitations regarding time since RFT are presented with the results. Further investigations should ideally be prospective trials with an adequate follow-up period.

## 5. Conclusions

In our sampled population, we observed that inequalities in social determinants of health correspond to higher disability scores, indicating that poor living standards are a common phenomenon in those affected by leprosy-related disabilities. Accordingly, social determinants of health are not just indispensable regarding the risk of transmission and infection, nor to the probability of a successful cure, but also correlate with the disability status of the affected persons. Our results suggest that especially age and less living space are predictors of higher disabilities due to leprosy. Further research is needed to dissect the exact development of impairments after RFT in order to take targeted actions against disability deterioration.

## Figures and Tables

**Table 1:** Disability grading in leprosy.

Disability Grade	Eyes	Hands and Feet
0	No eye impairment due to leprosy; no evidence of visual loss	No sensory impairment, no visible impairment
1	Eye problems due to leprosy present (irregular blink), but no vision impaired (can read fingers at six-meter distance)	Anesthesia present, but no visible deformity or damage, including muscle weakness without clawing
2	Severe visual impairments (cannot read fingers at six-meter distance), lagophthalmos, uveitis, corneal opacities	Visible impairments present, including ulcers and atrophy

The three-grade disability grading system for persons affected by leprosy was adapted from Brandsma et al. [31]. The sum of disabilities for each eye and extremity determines the Eyes-Hands-Feet (EHF) Score.

**Table 2:** Demographic and disability information of leprosy-affected study participants.

Characteristic		Female		Male		Total	
**Study participants**		41	33.33%	82	66.67%	123	100.00%
**Mean Age** (range)	years	63	(40–80)	66	(27–95)	65	(27–95)
**Social Class**							
Scheduled Tribe		2	4.88%	8	9.76%	10	8.13%
Scheduled Caste		13	31.71%	25	30.49%	38	30.89%
Most Backward Class		20	48.78%	32	39.02%	52	42.28%
Other Backward Class		6	14.63%	17	20.73%	23	18.70%
Forward Class		0	0.00%	0	0.00%	0	0.00%
**Literacy**							
Literate		4	9.76%	19	23.17%	23	18.70%
Illiterate		37	90.24%	63	76.83%	100	81.30%
**Average monthly income** (range)	INR	1444.88	(0–15,000)	1156.10	(0–6800)	1252.36	(0–15,000)
<1000 INR		3	7.32%	8	9.76%	11	8.94%
1000 INR		31	75.61%	61	74.39%	92	74.80%
>1000 INR		7	17.07%	13	15.85%	20	16.26%
**Employment Status**							
Unemployed		29	70.73%	63	76.83%	92	74.80%
Working		12	29.27%	19	23.17%	31	25.20%
**Household Ownership Situation**							
Own House		29	70.73%	63	76.83%	92	74.80%
With Family Members		2	4.88%	8	9.76%	10	8.13%
Renting		4	9.76%	3	3.66%	7	5.69%
Government Leprosy Home		6	14.63%	5	6.10%	11	8.94%
Homeless		0	0.00%	3	3.66%	3	2.44%
**Disabilities—Eyes**							
Grade 0		35	85.37%	60	73.17%	100	81.30%
Grade 1		0	0.00%	0	0.00%	0	0.00%
Grade 2		6	14.63%	17	20.73%	23	18.70%
**Disabilities—Hands**							
Grade 0		3	7.32%	7	8.54%	10	8.13%
Grade 1		0	0.00%	7	8.54%	7	5.69%
Grade 2		38	92.68%	68	82.93%	106	86.18%
**Disabilities—Feet**							
Grade 0		0	0.00%	2	2.44%	2	1.63%
Grade 1		11	26.83%	11	13.41%	22	17.89%
Grade 2		30	73.17%	69	84.15%	99	80.49%

Demographic and disability information of leprosy-affected study participants in Salem, Tamil Nadu, India. INR—Indian rupee.

**Table 3:** Detailed disability grading of leprosy-affected study participants.

Characteristic	≤1000 INR/M		>1000 INR/M		Total	
*n*	103		20		123	
Grade 2 Disabilities						
Both eyes	10	9.71%	0	0.00%	10	8.13%
Both hands	71	68.93%	8	40.00%	79	64.23%
Both feet	69	66.99%	7	35.00%	76	61.79%
Combined Grade 2 Disabilities						
Both eyes and hands	8	7.77%	0	0.00%	8	6.50%
Both eyes and feet	9	8.74%	0	0.00%	9	7.32%
Both hands and feet	59	57.28%	4	20.00%	63	51.22%
Both eyes, hands and feet	8	7.77%	0	0.00%	8	6.50%
EHF Score						
Average (95% CI)	7.398 (6.984–7.813)		5.050 (3.840–6.260)		7.016 (6.595–7,437)	
<7	33	32.04%	14	70.00%	47	38.21%
≥7	70	67.96%	6	30.00%	76	61.79%

Detailed disability grading of study participants stratified according to income. Relative ratios are related to the respective subgroup, including a detailed view of the disabilities of each pair of interest, eyes, hands, and feet, and the quantity of combined Grade 2 disabilities. EHF score averages as well as stratification, with a cut-off at seven were included. INR/M—Indian rupee per month.

**Table 4:** Multiple linear regression analysis of factors associated with EHF scores in leprosy-affected study participants.

Significant Predictors	Standardized Beta	95% CI		*p*
		Lower	Upper	
Age	0.340	0.039	0.111	<0.001
Living space	−0.276	−0.041	−0.011	0.001

## Supplementary Materials

The following are available online at http://www.mdpi.com/1660-4601/15/12/2769/s1, File S1—Dataset; Checklist S2—STROBE Statement; File S3—Supplementary data.
