# Supplementary material for: Analysis of Social Determinants of Health and Disability Scores in Leprosy-Affected Persons in Salem, Tamil Nadu, India
Source: Int J Environ Res Public Health. 2018 Dec 6;15(12):2769. doi: 10.3390/ijerph15122769 (PMC6313506; doi:10.3390/ijerph15122769)
Supplement: Supplementary file 1 [file ijerph-15-02769-s001.zip › Checklist S2 - STROBE Statement.pdf]

Checklist S2. STROBE Statement—checklist of items that should be included in reports of cohort studies

	Item No	Recommendation
**Title and abstract**	1	(a) Indicate the study's design with a commonly used term in the title or the abstract<br>**Manuscript, Title, Abstract**
		(b) Provide in the abstract an informative and balanced summary of what was done and what was found<br>**Manuscript, Abstract**
**Introduction**		
Background/rationale	2	Explain the scientific background and rationale for the investigation being reported<br>**Manuscript, Introduction**
Objectives	3	State specific objectives, including any prespecified hypotheses<br>**Manuscript, Introduction, Paragraph 6**
**Methods**		
Study design	4	Present key elements of the study design early in the paper<br>**Manuscript, Materials and Methods, Data Collection & Data Analysis**
Setting	5	Describe the setting, locations, and relevant dates, including periods of recruitment, exposure, follow-up, and data collection<br>**Manuscript, Materials and Methods, Study Area & Study Population & Data Collection**
Participants	6	(a) Give the eligibility criteria, and the sources and methods of selection of participants. Describe methods of follow-up<br>**Manuscript, Materials and Methods, Study Population**
		(b) For matched studies, give matching criteria and number of exposed and unexposed<br>No matching was undertaken
Variables	7	Clearly define all outcomes, exposures, predictors, potential confounders, and effect modifiers. Give diagnostic criteria, if applicable<br>**Manuscript, Materials and Methods, Data Collection & Data analysis**
Data sources/ measurement	8*	For each variable of interest, give sources of data and details of methods of assessment (measurement). Describe comparability of assessment methods if there is more than one group<br>**Manuscript, Materials and Methods, Study Population & Data Collection**
Bias	9	Describe any efforts to address potential sources of bias<br>Not applicable
Study size	10	Explain how the study size was arrived at<br>**Manuscript, Materials and Methods, Data Collection & Figure 1**
Quantitative variables	11	Explain how quantitative variables were handled in the analyses. If applicable, describe which groupings were chosen and why<br>**Manuscript, Materials and Methods, Data Analysis**
Statistical methods	12	(a) Describe all statistical methods, including those used to control for confounding<br>**Manuscript, Materials and Methods, Data Analysis**
		(b) Describe any methods used to examine subgroups and interactions<br>**Manuscript, Materials and Methods, Data Analysis**
		(c) Explain how missing data were addressed<br>**Manuscript, Results, Regression Analysis, Paragraph 4**

		(d) If applicable, explain how loss to follow-up was addressed<br>Not applicable
		(e) Describe any sensitivity analysis<br>**Manuscript, Materials and Methods, Data Analysis**
**Results**		
Participants	13*	(a) Report numbers of individuals at each stage of study—eg numbers potentially eligible, examined for eligibility, confirmed eligible, included in the study, completing follow-up, and analysed<br>**Manuscript, Results, Demographical Data**
		(b) Give reasons for non-participation at each stage<br>**Manuscript, Materials and Methods, Figure 1**
		(c) Consider using a flow diagram<br>**Manuscript, Materials and Methods, Figure 1**
Descriptive data	14*	(a) Give characteristics of study participants (e.g., demographic, clinical, social) and information on exposures and potential confounders<br>**Manuscript, Results, Demographical Data & Table 2**
		(b) Indicate number of participants with missing data for each variable of interest<br>**Manuscript, Results, Regression Analysis, Paragraph 4**
		(c) Summarise follow-up time (eg, average and total amount)<br>Not applicable
Outcome data	15*	Report numbers of outcome events or summary measures over time<br>Not applicable
Main results	16	(a) Give unadjusted estimates and, if applicable, confounder-adjusted estimates and their precision (e.g., 95% confidence interval). Make clear which confounders were adjusted for and why they were included<br>**Manuscript, Results, Regression Analysis & Table 3**
		(b) Report category boundaries when continuous variables were categorized<br>**Manuscript, Results**
		(c) If relevant, consider translating estimates of relative risk into absolute risk for a meaningful time period<br>Not applicable
Other analyses	17	Report other analyses done—e.g., analyses of subgroups and interactions, and sensitivity analyses<br>Not applicable
**Discussion**		
Key results	18	Summarise key results with reference to study objectives<br>**Manuscript, Results**
Limitations	19	Discuss limitations of the study, taking into account sources of potential bias or imprecision. Discuss both direction and magnitude of any potential bias<br>**Manuscript, Discussion, Regression Analysis & Study Limitations**
Interpretation	20	Give a cautious overall interpretation of results considering objectives, limitations, multiplicity of analyses, results from similar studies, and other relevant evidence<br>**Manuscript, Discussion**
Generalisability	21	Discussion the generalisability (external validity) of study results<br>**Manuscript, Discussion & Conclusion**
**Other information**		

Funding	22	Give the source of funding and the role of the funders for the present study and, if applicable, for the original study on which the present article is based **Manuscript, Funding**

*Give information separately for exposed and unexposed groups.

Note: An Explanation and Elaboration article discusses each checklist item and gives methodological background and published examples of transparent reporting. The STROBE checklist is best used in conjunction with this article (freely available on the Web sites of PLoS Medicine at http://www.plosmedicine.org/, Annals of Internal Medicine at http://www.annals.org/, and Epidemiology at http://www.epidem.com/). Information on the STROBE Initiative is available at http://www.strobe-statement.org.
