# Supplementary material for: Analysis of Social Determinants of Health and Disability Scores in Leprosy-Affected Persons in Salem, Tamil Nadu, India
Source: Int J Environ Res Public Health. 2018 Dec 6;15(12):2769. doi: 10.3390/ijerph15122769 (PMC6313506; doi:10.3390/ijerph15122769)
Supplement: Supplementary file 1 [file ijerph-15-02769-s001.zip › File S3 - Supplementary data.pdf]

File S3. Supplementary data.

Table S1. Independent variables of regression analysis with Eyes-Hands-Feet (EHF) score as dependent variable in study participants disabled through leprosy.

Dependent Variable	Independent variables		Collinearity	
	beta	P	VIF	Tolerance
**Gender (0 female, 1 male)**	-0.009	0.912	1.033	0.968
**Age**	0.340	<0.001	1.000	1.000
**Time after RFT (years)**	0.118	0.174	1.109	0.902
**Income (INR/M)**	-0.078	0.360	1.060	0.944
**Living space (qm^2)**	-0.276	0.001	1.000	1.000

VIF—Variance inflation factors; RFT—release from treatment; INR/M—Indian rupee per month.

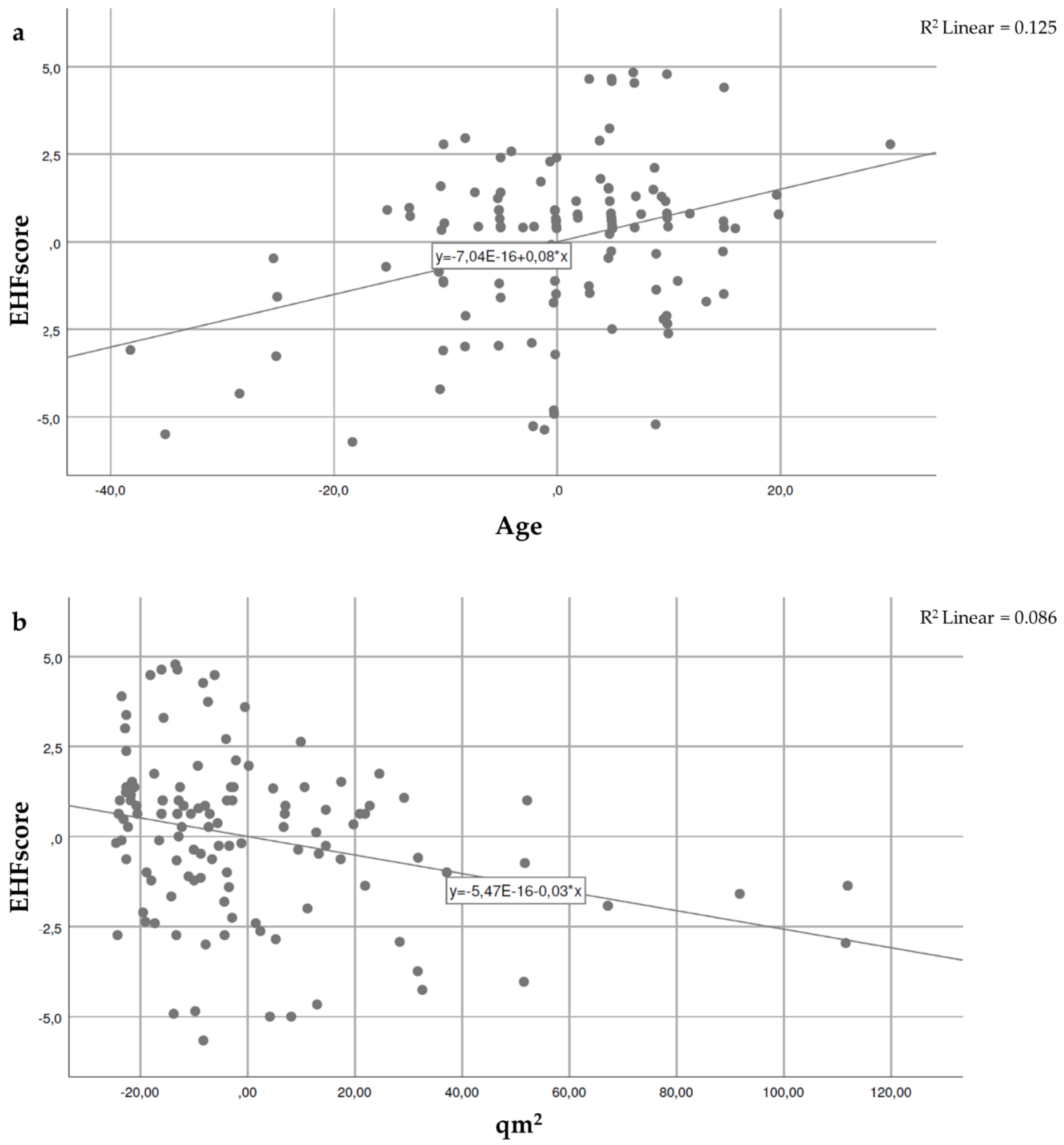

Figure S1. Linear regression plots to depict the relationship between the EHF score, **a)** age, and **b)** living space in leprosy affected study participants.
